# Modulation of Oxidative Stress, Inflammation, and Apoptosis and Restoration of Sirt1/Nrf2/HO-1 Signaling by Diosmin Protect Against Diabetes-Induced Testicular Damage in Rats

**DOI:** 10.3390/ijms27031268

**Published:** 2026-01-27

**Authors:** Saleem H. Aladaileh, Mohammad H. Abukhalil, Manal A. Alfwuaires, Abdulmohsen I. Algefare, Mohd Rasheeduddin Imran, Sayeeda Anjum, Shatha Alzahrani, Wael A. Alsubhi, Shaik Karimulla, Ayyad Hazzaa Al-Shammari

**Affiliations:** 1 Department of Pharmacy Practice, College of Pharmacy, University of Hafr Al Batin, Hafr Al Batin 39524, Saudi Arabia; dr.imran@uhb.edu.sa (M.R.I.); sayeedarasheed@uhb.edu.sa (S.A.); waalsubhi@uhb.edu.sa (W.A.A.); kshaik@uhb.edu.sa (S.K.); s2220001961@uhb.edu.sa (A.H.A.-S.); 2 Department of Biology, College of Science, Al-Hussein Bin Talal University, Ma’an 71111, Jordan; mabukhalil@ahu.edu.jo; 3 Department of Biological Sciences, Faculty of Science, King Faisal University, Al-Ahsa 31982, Saudi Arabia; malfwuaires@kfu.edu.sa (M.A.A.); aalgefare@kfu.edu.sa (A.I.A.); 4 Department of Clinical Laboratory Sciences, College of Applied Medical Sciences, Taif University, Taif 21944, Saudi Arabia; shalzahr@tu.edu.sa

**Keywords:** diabetes mellitus, testicular damage, inflammation, oxidative stress, diosmin, Sirt1, Nrf2

## Abstract

Diabetes mellitus (DM) is recognized as a major contributor to impaired testicular function and compromised male fertility. In the present study, the protective effects of the natural flavonoid diosmin (Dios) against diabetes-induced testicular injury were investigated using a rat model of streptozotocin (STZ)-induced diabetes. Diabetes was induced in rats via a single intraperitoneal injection of STZ at a dose of 50 mg/kg body weight. Dios was administered at doses of 25 and 50 mg/kg body weight for eight weeks. Diabetic rats displayed marked testicular dysfunction, evidenced by reduced serum testosterone levels, deteriorated sperm parameters, and pronounced histopathological alterations in testicular tissues. Biochemical analysis revealed elevated levels of oxidative stress markers, including malondialdehyde and protein carbonyls, along with decreased levels of reduced glutathione and diminished activities of catalase and superoxide dismutase in the testicular tissues. Furthermore, diabetes exacerbated testicular inflammation, as indicated by increased immunoexpression of NF-κB p65 and levels of pro-inflammatory cytokines. Likewise, diabetes induced testicular apoptosis, demonstrated by increased Bax and caspase-3 levels and decreased Bcl-2 levels. Treatment of diabetic rats with Dios significantly attenuated sperm parameters and testicular architecture and mitigated oxidative stress, inflammatory responses, and apoptotic cell death. Additionally, Dios enhanced antioxidant defense mechanisms and restored the Sirt1/Nrf2/HO-1 signaling pathway in the testicular tissues of diabetic rats. These results suggest that Dios may serve as an adjuvant therapeutic agent for diabetes-associated testicular dysfunction.

## 1. Introduction

Diabetes mellitus (DM) is associated with a substantial impairment of the male reproductive system, leading to testicular dysfunction and an elevated risk of infertility [1,2,3]. With the rising global prevalence of diabetes, increasing attention has been directed towards its effects on male reproductive functions, including spermatogenesis, hormonal regulation and the structural integrity of the blood-testis barrier (BTB) [1]. The key pathogenic mechanisms underlying hyperglycemia-induced testicular injury include, but not limited to, oxidative stress, chronic inflammation, and apoptosis, which collectively contribute to testicular dysfunction [1,3,4,5]. Hyperglycemia induces oxidative stress in the testes due to the imbalance between the excessive generation of reactive oxygen species (ROS) and insufficient antioxidative defenses. This oxidative stress damages cellular macromolecules, including proteins, lipids, and DNA, ultimately triggering apoptosis and compromising sperm quality [4,6]. Elevated ROS levels promote chronic inflammation, which further aggravates testicular injury [7,8]. Proinflammatory cytokines disrupt the physiological functions of testicular cells, impairing spermatogenesis [1,9,10]. Additionally, ROS and inflammatory mediators can initiate intracellular signaling pathways that activate caspases, leading to programmed germ cell death. Collectively, these molecular disturbances contribute to the structural and functional deterioration of the testes in diabetic males [1,11,12]. Furthermore, hormonal alterations, namely reduced testosterone levels, are often observed in diabetic males, in association with impaired spermatogenesis and the overall reproductive function [13,14]. Given the rising prevalence of diabetes and its harmful impact on male reproductive health, there is an urgent need for effective therapeutic interventions.

Multiple lines of evidence suggest that natural compounds can significantly restore testicular function by alleviating oxidative damage and enhancing antioxidant potential [5,10,12,15]. Among the natural products, flavonoids have been widely investigated for the treatment of male reproductive system dysfunction since they have strong antioxidant and anti-inflammatory actions [16]. It has been shown that flavonoids can mitigate ROS overproduction and oxidative tissue injury by improving antioxidant defense mechanisms and inhibiting nuclear factor-kappa B (NF-κB) signaling pathway and pro-inflammatory cytokines production [10,16,17,18,19,20]. In addition to their classical antioxidant and anti-inflammatory actions, flavonoids are recognized as active regulators of signaling pathways that control apoptosis, survival signaling, and transcriptional networks [21,22]. Several studies indicated that flavonoids, rather than acting solely as nonspecific redox scavengers, interact with key molecular signaling pathways, leading to coordinated regulation of cell cycle arrest, mitochondrial dysfunction, caspase activation, and stress-adaptive gene expression [21]. For example, flavonoids have been reported to activate the silent information regulator 1 (Sirt1) and nuclear factor erythroid 2-related factor 2 (Nrf2) [10,23,24], which are involved in maintaining the Leydig cell antioxidant environment and steroid production [25]. Diosmin (Dios), a flavonoid glycoside naturally present in citrus fruits, is recognized for its tissue protective potential and its ability to ameliorate various pathophysiological disruptions [26,27]. Several pharmacological activities have been identified for Dios, such as antioxidant, anti-diabetic, anti-inflammatory, and testicular protective effects [19,26,27]. The antioxidant and anti-inflammatory effects of Dios were demonstrated by its protective effects against doxorubicin-induced testicular injury [19]. Dios has also been shown to exert protective effects against tramadol-related reproductive impairment in male rats [28]. In addition, Dios has been reported to prevent cisplatin-evoked prostate and seminal vesicle injury by restoring redox imbalance and inhibiting apoptosis [29]. In type 2 diabetic rats, Dios was found to exert cardioprotective effects by enhancing antioxidant potential, modulating inflammatory pathways, and mitigating apoptotic cell death associated with diabetes-induced cardiac injury [30]. Dios was found to modulate NF-κB pathway and decrease oxidative stress markers in the kidney of alloxan-induced diabetic rats [31].

Extensive research highlights Dios’s involvement in modulating Sirt1 and Nrf2/heme oxygenase-1 (HO-1) signaling, which are key pathways involved in cellular defense mechanisms [18,32,33]. The histone deacetylase Sirt1 improves cellular resistance to oxidative stress and promotes Nrf2-mediated upregulation of antioxidant gene expressions such as HO-1, thereby strengthening the body’s intrinsic defense against oxidative and inflammatory damages [34,35,36,37]. The Nrf2/HO-1 signaling pathway is crucial in protecting against oxidative stress-related tissue damage by modulating inflammation, mitochondrial damage, and apoptosis [38,39]. Indeed, Sirt1 and Nrf2/HO-1 signaling has emerged as a promising target in the management of diabetes-related tissue damage [40,41,42,43,44]. Therefore, this study aimed to investigate the protective effects of Dios against diabetes-induced testicular injury by evaluating oxidative stress, inflammation, and apoptosis, with a particular focus on the Sirt1 and Nrf2/HO-1 signaling in a rat model streptozotocin (STZ)-induced diabetes. This investigation provides critical insights into Dios’s potential development as a potential protective tool for diabetic complications, especially in the context of male reproductive health.

## 2. Results

### 2.1. Impact of Dios on Metabolic Parameters and Body Weight in the Diabetic Rats

**Figure 1:**
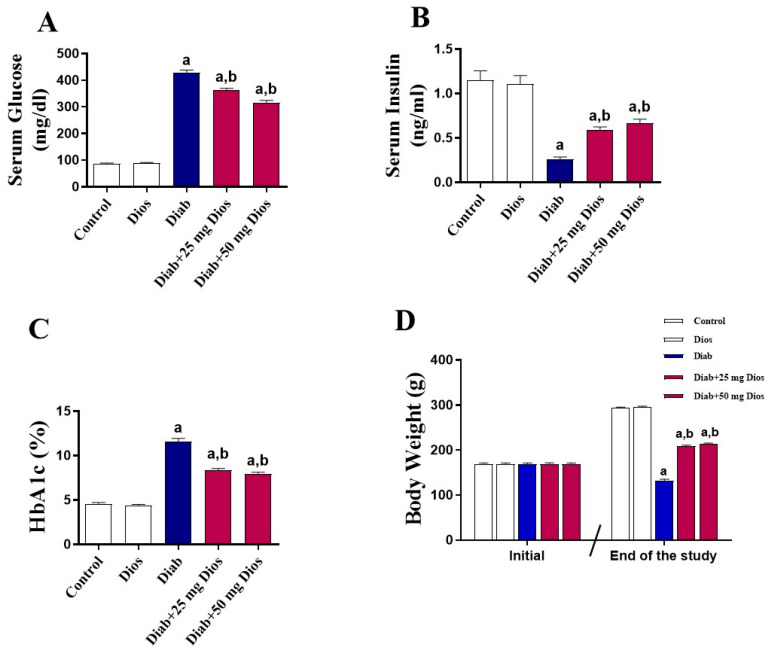
The impact of Dios on glucose, insulin, and HbA1c levels and body weight in diabetic rats. Dios treatment attenuated (**A**) glucose, (**B**) insulin, and (**C**) HbA1c levels and (**D**) body weight loss in diabetic rats. Results are presented as mean (±SEM, n = 8). (a) *p* < 0.05 vs. control; (b) *p* < 0.05 vs. Diab.

Induction of diabetes by STZ led to a significant (*p* < 0.05) elevation in serum glucose levels and HbA1c levels, along with a marked reduction in serum insulin levels and body weight when compared with control rats (Figure 1A–D). These diabetes-induced changes in serum glucose, HbA1c, and serum insulin levels and body weight were markedly improved when diabetic rats were treated with Dios (Figure 1A–D). No significant changes in these parameters were found in normal rats treated with Dios.

### 2.2. Effect of Dios on Sperm Parameters, Testosterone Levels, and Testicular Damage

**Figure 2:**
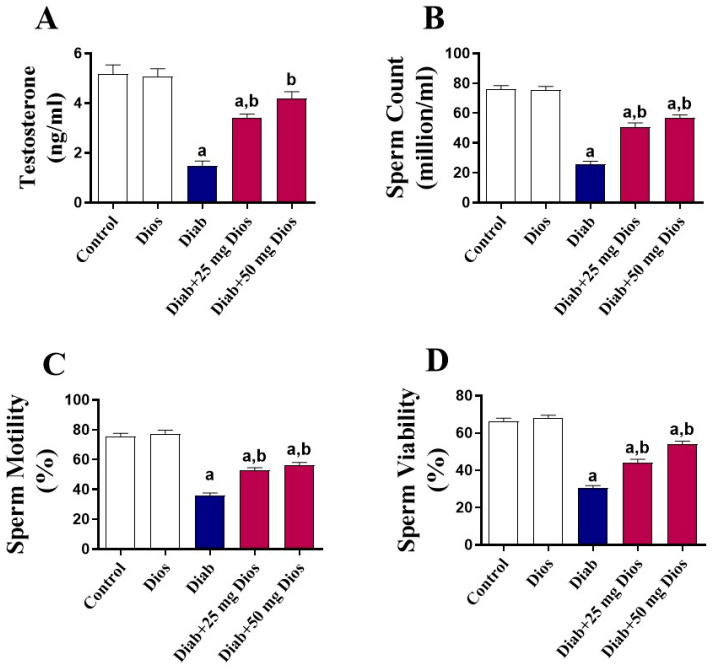
Effect of Dios on testosterone levels and sperm parameters in diabetic rats. Dios ameliorated (**A**) serum testosterone levels, (**B**) sperm count, (**C**) sperm motility, and (**D**) sperm viability in diabetic rats. Results are presented as mean (±SEM, n = 8). (a) *p* < 0.05 vs. control; (b) *p* < 0.05 vs. Diab.

Diabetes caused a significant (*p* < 0.05) reduction in serum testosterone levels (Figure 2A) and sperm parameters, including sperm count, motility, and viability (Figure 2B–D) when compared with control rats. These alterations were significantly (*p* < 0.05) attenuated when diabetic rats were treated with Dios (Figure 2A–D). No significant changes were observed in normal rats treated with Dios.

**Figure 3:**
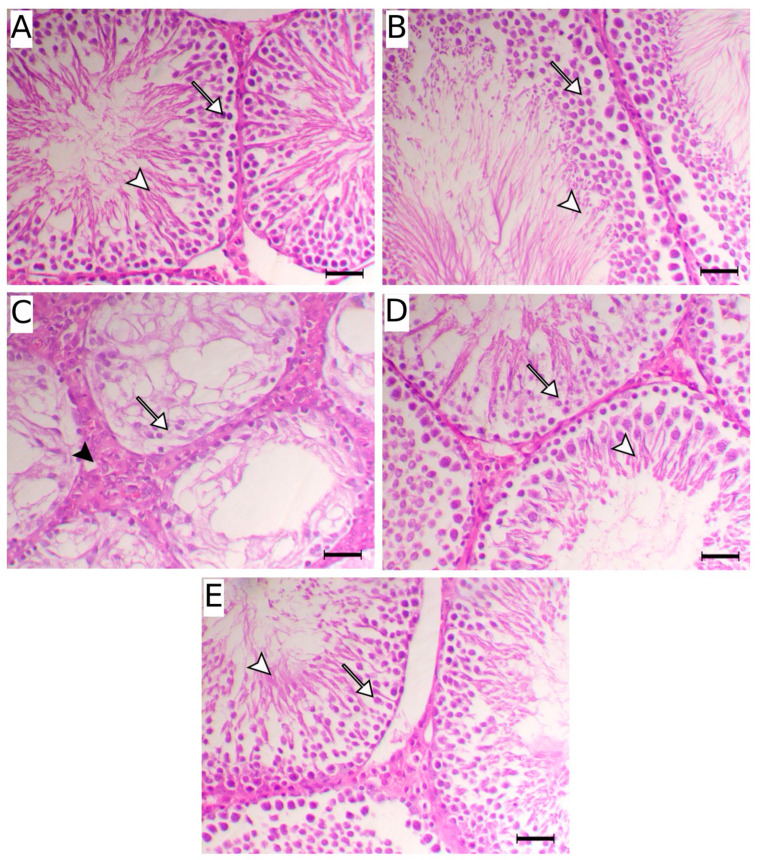
The diabetes-induced histological changes are attenuated by Dios. Histological examination of H&E-stained testicular sections of (**A**) control and (**B**) Dios-treated non-diabetic rats depicted normal seminiferous tubules lined with normal spermatogenic cells (white arrow) and free sperms within their lumen (white arrowhead). (**C**) H&E-stained testicular sections of diabetic rats depicted severe testicular atrophy associated with severe necrosis of the germinal epithelium (white arrow) and marked thickening of the basement membrane (black arrowhead). (**D**) H&E-stained testicular sections of diabetic rats treated with 25 mg Dios showed decreased degenerative changes within the spermatogenic cells (white arrow) with noticeable increase in the free luminal spermatocytes (white arrowhead). (**E**) H&E-stained testicular sections of diabetic rats treated with 50 mg Dios showed markedly attenuated degenerative changes within the spermatogenic cells (white arrow) with marked increase in normal spermatocytes (white arrowhead). Scale bar = 50 µm.

Moreover, H&E-stained sections of testicular tissues of control (Figure 3A) and non-diabetic rats treated with Dios (Figure 3B) illustrated intact seminiferous tubules with intact spermatogenic cells and mature sperm within the lumen. Diabetes induced several pathological changes, including severe testicular atrophy associated with severe necrosis of the germinal epithelium and marked thickening of the basement membrane (Figure 3C). These pathological alterations were ameliorated in Dios-treated diabetic rats (Figure 3D,E).

### 2.3. Mitigation of Oxidative Stress in the Testes of Diabetic Rats by Dios

**Figure 4:**
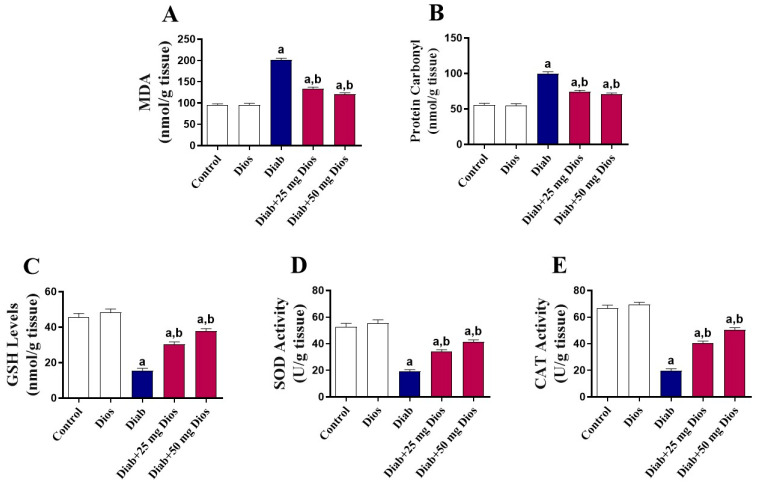
Dios alleviates diabetes-induced testicular oxidative stress in rats. Results of (**A**) MDA, (**B**) protein carbonyls, and (**C**) GSH levels and (**D**) SOD and (**E**) CAT activities in the testis are shown as mean (±SEM, n = 8). (a) *p* < 0.05 vs. control; (b) *p* < 0.05 vs. Diab.

Diabetes induction led to profound oxidative stress in testicular tissues, characterized by significantly (*p* < 0.05) increased levels of lipid peroxidation product, malondialdehyde (MDA) (Figure 4A) and protein carbaryl (Figure 4B) compared to control rats. These results were concordant with significantly (*p* < 0.05) decreased reduced glutathione (GSH) levels (Figure 4C) and superoxide dismutase (SOD) (Figure 4D) and catalase (CAT) (Figure 4E) activities in testicular tissue. These deleterious effects in the testicular tissues of diabetic rats were significantly (*p* < 0.05) attenuated by Dios treatment (Figure 4A–E). There was no effect of Dios on these parameters in normal rats.

### 2.4. Diabetes-Induced Testicular Inflammatory Response Is Attenuated by Dios

**Figure 5:**
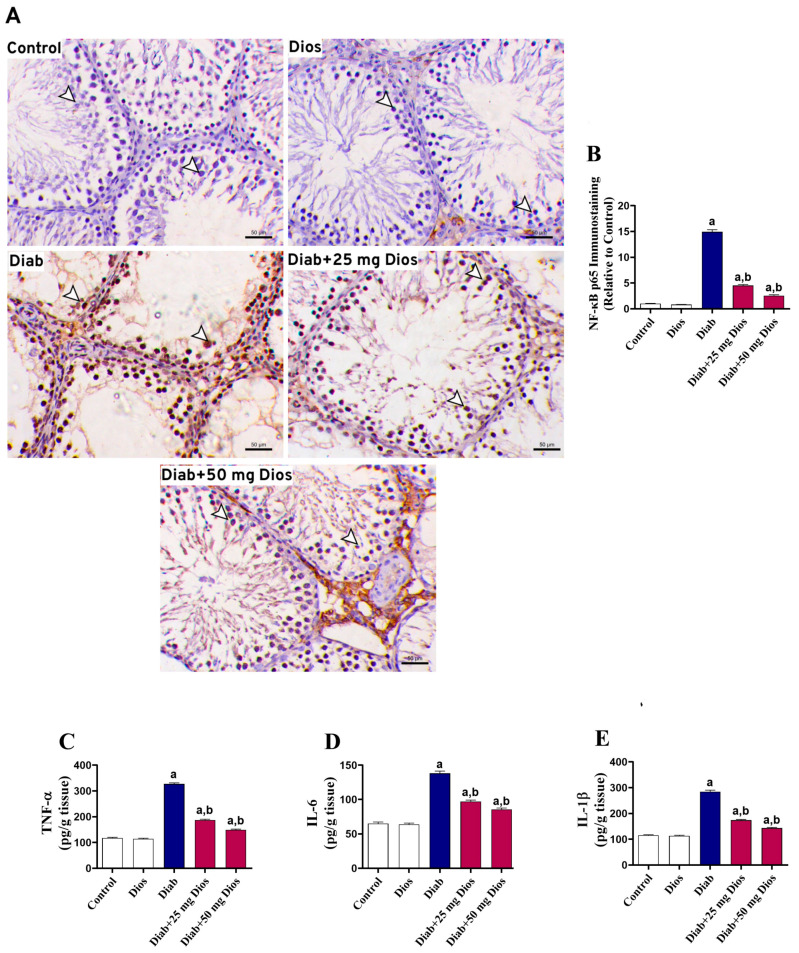
Dios attenuates diabetes-induced inflammation in the testis. (**A**) Representative immunostaining images of NF-κB p65 in the testicular tissues, where diabetic rats showed marked nuclear expression of NF-κB p65 within the spermatogenic cells (arrowheads) (IHC; Scale bar, 50 μm). (**B**) Immunostaining quantification of NF-κB p65 expressed relative to the control group (Mean ±SEM, n = 3). (**C**–**E**) Results of (**B**) TNF-α, (**C**) IL-6, and (**D**) IL-1β levels in the testicular tissues are shown as mean (±SEM, n = 8). (a) *p* < 0.05 vs. control; (b) *p* < 0.05 vs. Diab.

Immunoexpression levels of NF-κB p65 (Figure 5A,B) and levels of proinflammatory cytokines, including tumor necrosis factor-α (TNF-α), interleukin-6 (IL-6) and IL-1β (Figure 5C–E), were significantly (*p* < 0.05) elevated in the testicular tissues of diabetic rats. These protein levels were significantly (*p* < 0.05) decreased in Dios-treated diabetic rats, indicating an alleviated inflammatory response (Figure 5A–E). No significant changes in these parameters were found in normal rats treated with Dios.

### 2.5. Alleviation of Testicular Apoptosis in Diabetic Rats by Dios

**Figure 6:**
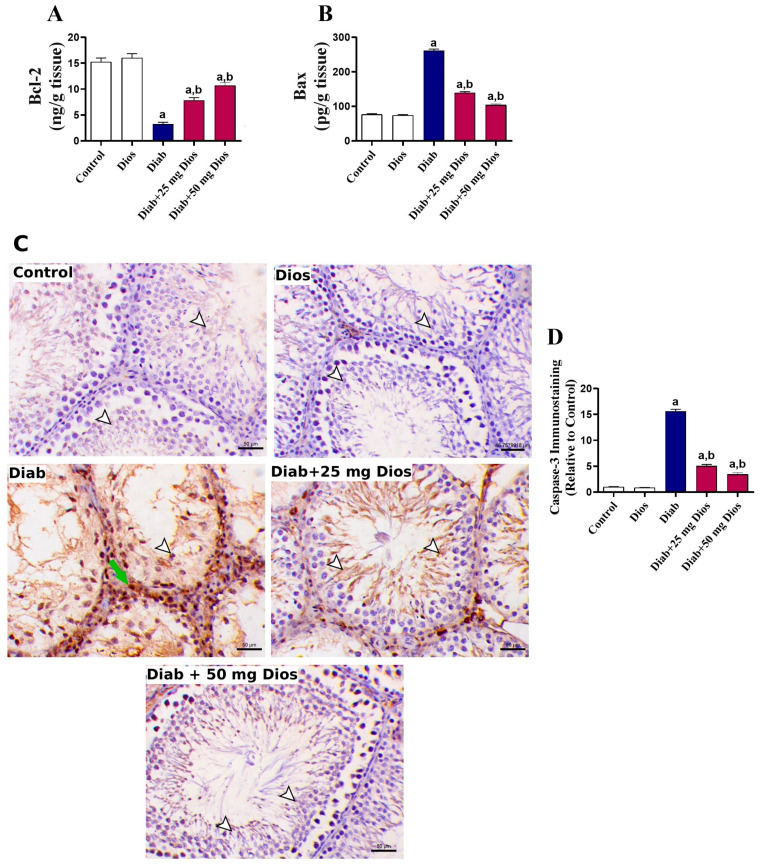
Dios attenuates diabetes-induced testicular apoptosis. Results of (**A**) Bcl-2 and (**B**) Bax in the testicular tissues are shown as mean (±SEM, n = 8). (**C**) Representative immunostaining images of caspase-3, where diabetic rats showed increased expression of caspase-3 within both spermatogenic cells (arrowhead) and the interstitial cells (green arrow) (IHC; Scale bar, 50 μm). (**D**) Immunostaining quantification of caspase-3 expressed relative to the control group (n = 3). Data are presented as means ± SEM. (a) *p* < 0.05 vs. control; (b) *p* < 0.05 vs. Diab.

There was a significant (*p* < 0.05) decrease in Bcl-2 levels (Figure 6A), along with increase in Bax levels (Figure 6B) and caspase-3 immunoexpression levels (Figure 6C,D) in the testicular tissues of diabetic rats compared to control rats. Treatment with Dios significantly (*p* < 0.05) attenuated the levels of these apoptosis-related proteins in the testicular tissues of diabetic rats. There was no effect on these parameters in normal rats treated with Dios.

### 2.6. Dios Modulates Sirt1 and Nrf2/HO-1 in Diabetic Rat Testes

**Figure 7:**
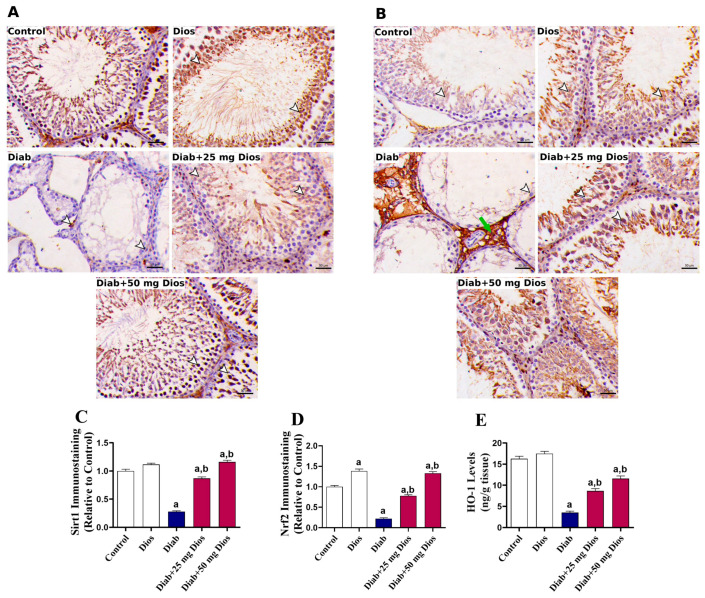
Dios modulates Sirt1 and Nrf2/HO-1 signaling in the testes of diabetic rats. (**A**) Representative immunostaining images of Sirt1 in testicular tissues are shown (IHC; Scale bar, 50 μm). (**B**) Representative immunostaining images of Nrf2 in testicular tissues are shown. Arrowheads indicate spermatogenic cells, while the green arrow indicates the interstitial cells. (**C**,**D**) Immunostaining quantification of Sirt1 and Nrf2, respectively, expressed relative to the control group (Mean ±SEM, n = 3). (**E**) Results of HO-1 levels in testicular tissues are shown as mean (±SEM, n = 8). (a) *p* < 0.05 vs. control; (b) *p* < 0.05 vs. Diab.

STZ-induced diabetes led to a significant (*p* < 0.05) decrease in Sirt1 (Figure 7A,C) and Nrf2 expression (Figure 7B,D), with a significant (*p* < 0.05) decrease in HO-1 levels (Figure 7E) in the testicular tissues of diabetic rats. These levels were significantly (*p* < 0.05) restored in Dios-administered diabetic rats (Figure 7A–E).

## 3. Discussion

Extensive evidence suggests that cellular oxidative imbalance and activation of inflammatory response and apoptosis are key factors for the onset of testicular injury in diabetes, ultimately leading to testicular dysfunction [10,11,12,13,15,44,45]. The Sirt1/Nrf2 pathway is integral in mitigating these effects in diabetes-related tissue damage by upregulating antioxidant enzymes and suppressing pro-inflammatory signals [40,41,42,43,44]. Dios is a flavonoid glycoside predominantly present in citrus fruits and is widely studied for its health-promoting effects [26,27]. Herein, we report that Dios treatment attenuates diabetes-induced testicular tissue damage by decreasing oxidative stress, inflammatory responses, and apoptosis while restoring Sirt1/Nrf2/HO-1 pathway.

Consistent with multiple reports [46,47,48,49], rats with STZ-induced diabetes exhibited a notable decrease in body weight, increase in glucose and HbA1c levels, and decrease in insulin levels. Diabetes induction by STZ is widely accepted as an experimental model to understand the pathogenesis of DM and to assess potential therapeutic interventions [46,47,49,50]. STZ destroys pancreatic β-cells, leading to impaired insulin secretion and subsequent elevated blood glucose levels [51]. Once inside the pancreatic β-cells, STZ causes DNA alkylation and excessive ROS production. This leads to DNA damage, activation of poly (ADP-ribose) polymerase (PARP), and subsequent depletion of intracellular NAD^+^ and ATP, ultimately resulting in β-cell destruction and a marked reduction in insulin secretion [51,52]. The current study showed that Dios attenuated body weight loss and glucose, HbA1c, and insulin levels in STZ-injected rats. It has been reported that Dios lowered glucose and HbA1c levels while increased plasma insulin concentrations in diabetic rats [53]. Dios was able to attenuate the activities of hepatic enzymes essential for glucose metabolism, including glucose-6-phosphate dehydrogenase, glucose-6-phosphatase, hexokinase, and fructose 1,6-biphosphatase [53]. In another study conducted on STZ-induced diabetic rats, Dios was shown to enhance GLUT 4 (glucose transporter subtype 4) expression levels in soleus muscle and to attenuate hepatic gluconeogenesis [54].

The diabetic state is often accompanied by increased oxidative stress, which significantly contributes to testicular impairment [1,4,5,8,11,12,14,15,44]. Consistent with multiple reports [5,15,45,49,55], our results showed that diabetes induction led to a substantial elevation in MDA and protein carbaryl levels, accompanied by a pronounced reduction in GSH content and diminished SOD and CAT activities in testicular tissues. These changes indicate a shift toward a pro-oxidant signaling state that favors cellular dysfunction and stress-responsive pathways in diabetic testes. Indeed, hyperglycemia, a hallmark of DM, can induce ROS generation through several mechanisms, including mitochondrial dysfunction, stimulation of NADPH oxidase activity along with generation of advanced glycation end products (AGEs) [1,56]. Increased ROS generation leads to damage in various cellular components, ultimately impairing testicular function [4,6]. It has been reported that cellular oxidative damage impairs mitochondrial function, leading to continuous ROS formation and a decline in ATP generation, triggering apoptosis and eventually contributing to germ cell loss [8,57]. Moreover, increased oxidative imbalance within the testicular tissue interferes with spermatogenesis, elevates sperm abnormalities, causes loss of germ cells via apoptosis, and decreases the ability of Leydig cells to produce testosterone [58,59]. The redox imbalance in this study was accompanied by a marked reduction in sperm parameters, including their count, motility and viability, along with a significant decline in serum testosterone levels in diabetic rats. Moreover, diabetes induction resulted in distinct pathological alterations within the testicular tissue, including severe testicular atrophy associated with severe necrosis of the germinal epithelium and marked thickening of the basement membrane. Therefore, therapeutic interventions targeting oxidative stress can potentially mitigate these effects and improve male reproductive health. In our findings, treatment of diabetic rats with Dios mitigated MDA and protein carbaryl levels and restored GSH content and SOD and CAT activities in testicular tissue. Previous studies have established the antioxidant potential of Dios in several disease models, including cisplatin-induced prostrate and seminal vesicle damage [29], doxorubicin-mediated testicular toxicity [19], and gentamicin-triggered nephrotoxicity [18] in animals, where Dios ameliorated oxidative damage and enhanced antioxidant defenses. Dios was able to attenuate histopathological changes in testicular tissues and the change in serum testosterone and sperm parameters in doxorubicin-injected male Wistar rats [19].

Persistent hyperglycemia and oxidative stress are known to activate inflammatory pathways, including the NF-κB pathway, which elevates the expression of pro-inflammatory cytokines, further impairing testicular function [11,60,61]. Inflammation exacerbates testicular damage and impairs Leydig cell function, reducing testosterone synthesis [7]. Moreover, hyperglycemia and inflammation can compromise the integrity of the BTB, allowing harmful substances to enter the seminiferous tubules and disrupt sperm development [1]. Furthermore, enhanced inflammatory response and oxidative damage can initiate apoptotic signaling, thereby perpetuating the development of testicular damage [7,11,62]. Indeed, alteration of Bax/Bcl-2 ratio has been shown to be an essential gateway to promote mitochondrial membrane potential dissipation and cytochrome c release, thereby committing cells to intrinsic apoptosis and subsequently activating the execution phase of caspase-3-dependent apoptotic cell death [63,64]. Increased apoptosis reduces sperm production and decreases testicular function and fertility [65]. In line with earlier findings [10,12,15,44,49,60,61], diabetic testicular tissues were characterized by increased inflammation, as evidenced by elevated NF-κB p65 expression and increased levels of TNF-α, IL-1β, and IL-6. Moreover, testicular tissues of diabetic rats showed enhanced apoptotic signaling, indicated by reduced Bcl-2 levels and increased levels of Bax and immunoexpression of caspase-3. Importantly, mitigation of oxidative stress, inflammation, and subsequent apoptosis represents a potential therapeutic strategy to alleviate diabetes-induced testicular injury and preserve male fertility. Herein, Dios treatment mitigated diabetes-related inflammatory and apoptotic changes in the testicular tissues by modulating inflammatory mediators, including NF-κB p65, TNF-α, IL-1β, and IL-6, together with apoptotic regulatory proteins, including Bcl-2, Bax, and caspase-3. In a non-sterile inflammatory pain and peritonitis model triggered by lipopolysaccharide (LPS), Dios reduced NF-κB signaling and lowered TNF-α, IL-1β, and IL-6 production in mice [66]. Moreover, Dios attenuated NF-κB signaling, downregulated NLRP3 inflammasome expression, and decreased the production of TNF-α, IL-1β, and IL-6 in the prefrontal cortex of LPS-treated mice displaying depression-like symptoms [20]. Dios has also been reported to reduce STZ-induced cardiac inflammatory responses and apoptotic signaling in rats by modulating TNF-α, IL-1β, Bcl-2, Bax, and caspases-3/9 [30]. Dios’s protective role on diabetes-associated testicular impairment is mechanistically attributed, at least in part, to its ability to enhance redox balance, mitigate NF-κB-mediated inflammatory signaling, and modulate key apoptotic markers, such as Bcl-2, Bax, and caspase-3.

Compelling in vivo evidence implies that modulating Sirt 1 and Nrf2/HO-1 pathway plays crucial role in protecting testicular tissue in the diabetic milieu [45,67,68]. Herein, the current investigation also evaluated the influence of Dios on the immunoexpression levels of Sirt1 and Nrf2 along with HO-1 content in testicular tissues of rats with and without diabetes. Consistent with previous reports [44,45,55,67,68], testicular tissues of diabetic rats showed diminished Sirt1 and Nrf2 immunoexpression with a decline in HO-1 content. Sirt1 plays a protective role against tissue injury by mitigating oxidative imbalance, inflammatory signaling, and apoptotic process by upregulating the expression of antioxidant enzymes and deacetylating many substrates, including the p65 component of NF-κB and the p53 protein [35,69,70] and helps to regulate spermatogenesis by affecting key functional processes of germ cells as well as Sertoli cells and Leydig cells [71,72]. It has been reported that Sirt1 deficiency resulted in less number of fully developed spermatozoa in adult mice, decreased number of spermatogenic stem cells in male embryos, and heightened DNA instability in male germ cells [73]. It has been shown that Sirt1 can increase the activation of Nrf2 and its downstream key antioxidant gene, HO-1, by deacetylating the transcription coactivator peroxisome proliferator-activated receptor gamma coactivator 1-alpha (PGC-1α) [74]. In turn, Nrf2 signaling pathway improves cellular antioxidant capacity, contributing to tissue protection by counteracting oxidative stress and dampening inflammatory responses [38,39]. It has been reported that activation of the testicular Sirt1/Nrf2/HO-1 cascade, among others, by dapagliflozin can protect against oxidative tissue injury and testicular dysfunction in cadmium-treated rats [75]. Indeed, Nrf2 regulates the expression of numerous antioxidant genes, including HO-1 and additional enzymes involved in antioxidant defense [38]. Nrf2 also limits the activation of NF-κB and the subsequent inflammatory cascade by blocking proteasomal degradation of IκB-α and by reducing the ROS-induced oxidative stress inside the cells [39,76]. The importance of Nrf2 in male fertility is emphasized according to the earlier research involving male Nrf2-knockout mice showing that its deficiency resulted in impaired spermatogenesis and fertility in an age-dependent manner, increased lipid peroxidation in the testis and epididymis, enhanced apoptosis of testicular germ cells, and reduced antioxidant levels compared with wild-type males [77]. Therefore, therapeutic strategies aimed at enhancing Sirt1 and Nrf2/HO-1 hold promise for preventing and treating diabetes-related testicular damage. Herein, treatment of diabetic rats with Dios restored the levels of Sirt1, Nrf2, and HO-1 in the testicular tissues. Accordingly, Dios modulated Nrf2 and NF-κB signaling to inhibit oxidative stress and inflammatory reaction in ulcerative colitis model [33]. Dios also modulated Nrf2/HO-1 and protected against gentamicin-induced nephrotoxicity in rats [18]. A prior study showed that Dios effectively inhibited NF-κB signaling, activated Nrf2 and antioxidant defense systems, and mitigated pro-inflammatory cytokines to alleviate rheumatoid arthritis induced by complete Freund’s adjuvant in rats [78]. In addition, Dios in combination with perindopril could effectively attenuate oxidative stress, inflammation, and apoptosis through inhibiting NF-κB and enhancing the levels of Sirt1, Nrf2, and HO-1, thereby offering protection against amikacin-induced nephrotoxicity in rats [32]. Therefore, the ability of Dios to attenuate diabetes-induced oxidative tissue injury and inflammation in the testes appears to be primarily linked to its capacity to restore Sirt1, Nrf2/HO-1 signaling pathway.

Although Dios exhibited marked biological activity in this study, flavonoids, including Dios, are generally characterized by limited bioavailability, variability in potency, and the need for large-scale clinical validation [79,80]. Recent nanotechnology-based therapeutic strategies have been shown to enhance the pharmacological and biochemical utility of flavonoids in a variety of diseases [81]. Although such strategies are beyond the scope of this study, they could be a promising avenue to improve the translational efficiency of Dios in future investigations.

## 4. Materials and Methods

### 4.1. Animals

Experimental procedures in this study adhered to ARRIVE guidelines (Animal Research: Reporting of *In Vivo* Experiments). Animal procedures were also consistent with the National Institutes of Health (NIH) for the Care and Use of Laboratory Animals and were approved by the Research Ethics Committee of King Faisal University (KFU-REC-2025-ETHICS3591). Adult male Wistar rats (160–180 g) were used in the study and maintained under a controlled laboratory environment with a controlled 12 h cycle of light and dark periods, regulated temperature, and stable humidity. All animals were acclimatized for one week prior to the experimental procedures and were allowed to feed and drink freely on standard diet.

### 4.2. Induction of Diabetes in Rats

Male Wistar rats were rendered diabetic via a single intraperitoneal (i.p.) injection of STZ at a dose of 50 mg/kg body weight [46]. A freshly prepared solution of STZ (Glentham Life Sciences, Corsham, UK) was made using 0.1 M citrate buffer adjusted to pH 4.5. The diabetic state was established by assessing fasting glucose levels with a glucometer seven days following STZ injection and the rats that showed glucose levels ≥ 250 mg/dl were classified as diabetic and subsequently enrolled in the study.

### 4.3. Study Design and Animal Grouping

Following acclimatization, the rats were randomly divided into five experimental groups (n = 8). The control group (I) consisted of non-diabetic rats receiving the vehicles. The Dios group (II) assigned non-diabetic rats that received Dios (50 mg/kg body weight, orally), prepared in dimethyl sulfoxide (DMSO). The diabetic group (III) included diabetic rats receiving DMSO orally. The Diab + 25 Dios group (IV) comprised diabetic rats treated with Dios at a dose of 25 mg/kg body weight, orally. The Diab + 50 Dios group (V) comprised diabetic rats treated with Dios at a dose of 50 mg/kg body weight, orally. The administered doses of Dios (Santa Cruz Biotechnology, Dallas, TX, USA) in the current study were determined based on earlier reports demonstrating anti-inflammatory and antioxidant effects of Dios [17,82].

After the completion of treatment period (8 weeks), all rats were anesthetized with a combination of ketamine and xylazine [100:10 mg/kg body weight, i.p.], and blood samples were collected via cardiac puncture. For biochemical assays, the serum was obtained by centrifugation and stored at −80 °C. Some blood samples from experimental groups were collected on tubes containing EDTA for the estimation of glycosylated hemoglobin (HbA1c). Testicular tissues were isolated from experimental rats and processed for the analysis of various parameters. From each pair of testes, one testis was treated with 10% neutral buffered formalin (NBF) for histological processing and immunohistochemical (IHC) analysis, while the other testis was homogenized and used for further biochemical assessments.

### 4.4. Sperm Preparation and Analysis

For sperm analysis, the sperm samples were taken from the cauda epididymis of male rats. Briefly, the cauda epididymis was carefully minced and immersed in 5 mL of pre-warmed phosphate-buffered saline (PBS). The sperm was then kept at 37 °C for 5 min to let sperm to swim out, creating a cloud suspension [49,83]. For sperm count, a 10 µL sample of sperm suspension was loaded into the hemocytometer, and the sperm number was counted under a light microscope at 200× magnification. The sperm count was expressed as millions of sperm per milliliter. Sperm motility was assessed microscopically by placing a drop of sperm suspension on a pre-warmed microscope slide and observing under a light microscope. Sperm motility was expressed as the percentage (%) of total motile sperms to total number of sperms. The percentage of sperm viability was determined using a stain exclusion method with eosin-nigrosin stain as previously described [83,84], where the viable sperm excluded the stain and appeared with white or light pink heads, while the dead sperm took up the dye and exhibited red or dark pink heads. Results were reported as the percentage (%) of viable sperm.

### 4.5. Biochemical Analysis

Levels of glucose in serum and HbA1c in blood were assessed using kits obtained from Spectrum Diagnostics (Al-Qalyubia Governorate, Cairo, Egypt). Serum levels of insulin were estimated using enzyme-linked immunosorbent assay (ELISA) kit provided by MyBioSource (San Diego, CA, USA) per the manufacturer’s instructions. Testosterone levels in serum were assessed using ELISA kit (CUSABIO, Houston, TX, USA) per the manufacturer’s instructions. Testicular tissue homogenates were used to measure the levels of MDA [85], protein carbonyl [86], and GSH [87] and activities of SOD [88] and CAT [89]. ELISA kits were used to assess the levels of testicular pro-inflammatory cytokines, including TNF-α (MyBioSource, San Diego, CA, USA) and IL-6 and IL-1β (CUSABIO, Houston, TX, USA) per the manufacturer’s instructions. Levels of HO-1, Bcl-2, and Bax in testicular tissues were assessed using ELISA kits provided by MyBioSource (San Diego, CA, USA) per the manufacturer’s instructions.

### 4.6. Histological and Immunohistochemical Analysis

Testicular samples preserved in 10% NBF were dehydrated, embedded in paraffin, sectioned at 5 μm thickness, and stained with hematoxylin and eosin (H&E) for histopathological examination. These tissue sections were examined under Leica DFC camera-equipped Leica microscope (Leica Microsystems, Wetzlar, Germany) to assess histological appearance and tissue damage of the testes [90].

The IHC analysis was conducted to evaluate the immunoexpression of NF-κB p65, caspase-3, Nrf2, and Sirt1 in testicular tissues. In brief, tissue sections were deparaffinized and exposed to citrate buffer solution (0.05 M, pH 6.8) to retrieve antigens. Hydrogen peroxidase (H_2_O_2_) was further added to the solution to inhibit intrinsic peroxidase activity. Subsequently, the mounted sections were exposed to primary antibodies and maintained at 4 °C overnight against Sirt1 at a dilution of 1:200 (GeneTex, Inc., Irvine, CA, USA), Nrf2 and caspase-3 (each at a dilution ratio 1:100) procured from Invitrogen (Waltham, MA, USA), and NF-κB p65 (used at a 1:100 dilution, Santa Cruz Biotechnology, Dallas, TX, USA). After washing with PBS, the tissue sections were exposed to the corresponding secondary antibodies (EnVision+^TM^ System Horseradish Peroxidase Labelled Polymer, Agilent Technologies, Inc., Santa Clara, CA, USA). Immunostaining was developed with diaminobenzidine (DAB), and the tissue sections were processed through Mayer’s hematoxylin countersting. Staining labeling indices of NF-κB p65 and caspase-3 were assessed as count of positive cells within 1000 total spermatogenic cells and expressed as a percentage, while staining intensity of Sirt1 and Nrf2 was determined through the area of positive expression using ImageJ software (Version 1.53t, NIH, Bethesda, MD, USA). Immunoexpression level from IHC staining was calculated and shown as relative to the control group.

### 4.7. Data Representation and Analysis

Data were presented as mean ± SEM. They were tested for normality and homogeneity of variances using the Shapiro–Wilk test and Levene’s test, respectively. GraphPad Prism 8 (GraphPad Inc., San Diego, CA, USA) was employed to perform one-way ANOVA followed by Tukey’s multiple comparison post hoc test to differentiate among experimental groups. Probability values of *p* < 0.05 were considered significant.

## 5. Conclusions

Our present study provides strong evidence that Dios attenuates diabetes-associated testicular dysfunction in rats. Dios attenuated testosterone and sperm parameters and reduced testicular oxidative stress, inflammatory responses, and apoptosis in diabetic rats. These protective actions of Dios were linked to the modulation of Sirt1 and Nrf2/HO-1 levels in the testis of diabetic rats. Thus, Dios might have a notable role in the prevention and treatment of diabetes-associated testicular complications and male reproductive dysfunction. Further investigations are required to elucidate the underlying mechanisms of Dios’s protective effects. 

## Data Availability

The original contributions presented in this study are included in the article. Further inquiries can be directed to the corresponding author.
